# T helper cells exhibit a dynamic and reversible 3′-UTR landscape

**DOI:** 10.1261/rna.079897.123

**Published:** 2024-04

**Authors:** Denis Seyres, Oliver Gorka, Ralf Schmidt, Romina Marone, Mihaela Zavolan, Lukas T. Jeker

**Affiliations:** 1 Department of Biomedicine, Basel University Hospital and University of Basel, CH-4031 Basel, Switzerland; 2 Transplantation Immunology and Nephrology, Basel University Hospital, CH-4031 Basel, Switzerland; 3 Computational and Systems Biology, Biozentrum, University of Basel, 4056 Basel, Switzerland; 4 Swiss Institute of Bioinformatics, Biozentrum, University of Basel, 4056 Basel, Switzerland

**Keywords:** alternative polyadenylation, posttranscriptional regulation, 3′-UTR usage, T helper cells, effector/memory T cells, naive T cells, proliferation

## Abstract

3′ untranslated regions (3′ UTRs) are critical elements of messenger RNAs, as they contain binding sites for RNA-binding proteins (RBPs) and microRNAs that affect various aspects of the RNA life cycle including transcript stability and cellular localization. In response to T cell receptor activation, T cells undergo massive expansion during the effector phase of the immune response and dynamically modify their 3′ UTRs. Whether this serves to directly regulate the abundance of specific mRNAs or is a secondary effect of proliferation remains unclear. To study 3′-UTR dynamics in T helper cells, we investigated division-dependent alternative polyadenylation (APA). In addition, we generated 3′ end UTR sequencing data from naive, activated, memory, and regulatory CD4^+^ T cells. 3′-UTR length changes were estimated using a nonnegative matrix factorization approach and were compared with those inferred from long-read PacBio sequencing. We found that APA events were transient and reverted after effector phase expansion. Using an orthogonal bulk RNA-seq data set, we did not find evidence of APA association with differential gene expression or transcript usage, indicating that APA has only a marginal effect on transcript abundance. 3′-UTR sequence analysis revealed conserved binding sites for T cell-relevant microRNAs and RBPs in the alternative 3′ UTRs. These results indicate that poly(A) site usage could play an important role in the control of cell fate decisions and homeostasis.

## INTRODUCTION

As part of the adaptive immune system, naive T helper cells emerge from the thymus to circulate through the body in search of their cognate antigen presented by a specialized antigen-presenting cell. During this phase, CD4**^+^** T cells are in a quiescent state, mainly dependent on signals conveyed by a T cell receptor (TCR): major-histocompatibility complex (MHC)-class II interaction and IL-7 (Surh and Sprent 2008). Upon encountering their cognate antigen and costimulatory signals, T cells start to proliferate and, in the presence of cytokines, can differentiate into effector T cell subsets with specialized roles (Ruterbusch et al. 2020; Dong 2021). Some T cell subsets promote inflammatory conditions, while regulatory T cells (Tregs) have a dampening, immunomodulatory role (Okeke and Uzonna 2019; Sakaguchi et al. 2020). Both the expansion and the differentiation phases are tightly regulated, on multiple molecular levels. While the transcriptional control has been widely investigated, posttranscriptional regulation is less well understood. By binding to the 3′ untranslated regions (3′ UTRs) of key target transcripts, thereby affecting their stability and protein expression, microRNAs (miRNAs) and RNA-binding proteins (RBPs) regulate many cellular processes, including the cell cycle, metabolism, and cell differentiation (Fu and Blackshear 2017; Bartel 2018; Rodríguez-Galán et al. 2018). Global shortening of 3′ UTRs has been observed in rapidly dividing cells, such as cancer cells and activated lymphocytes, suggesting a link between alternative polyadenylation (APA) and oncogenesis and lymphocyte differentiation (Sandberg et al. 2008; Mayr and Bartel 2009). Conversely, differentiation of embryonic stem cells is associated with the opposite trend of general 3′-UTR lengthening (Ji et al. 2009; Gruber and Zavolan 2019; Mitschka and Mayr 2022).

Previous work on human and mouse T cells has shown that activation is accompanied by a genome-wide trend toward increased usage of coding region-proximal polyadenylation sites, leading to shorter 3′ UTRs (Sandberg et al. 2008; Gruber et al. 2016). 3′-UTR remodeling did not lead to large changes in mRNA and protein levels (Gruber et al. 2016). However, subsequent studies revealed that the 3′-UTR isoform-specific RBP interactome can affect other processes such as the localization of the protein translated from the mRNA. One example is provided by CD47 isoforms, in which the 3′-UTR acts as a scaffold for the binding of the RBP HuR, which facilitates the localization of CD47 to the plasma membrane (Berkovits and Mayr 2015). In this project, we set out to determine when the dynamic remodeling of 3′ UTRs takes place in the helper T cell life cycle and what implication this process could have for T cell function. We generated 3′-UTR end sequencing data and performed pairwise comparisons of functionally annotated APA genes in naive, activated, and differentiated T cells. While 3′-UTR shortening events were found to be more frequent upon T cell activation, we identified a substantial number of lengthening events. To further validate the APA sites inferred by 3′ end sequencing, we also generated PacBio long-read sequencing data. While transcript coverage by PacBio reads was sparse, we found a meaningful overlap between the isoforms identified with this technology and by 3′ end sequencing. Next, we sought to determine whether the APA changes are reversible following T cell activation. We identified 1110 genes (Supplemental Table S2) associated with transient 3′-UTR length changes in T helper cells during acute activation and effector phase expansion, meaning that the cells eventually return to a naive state of polyadenylation site usage when re-entering a resting state as memory T cells. Using an orthogonal mRNA data set, we observed a limited effect of APA on gene expression and regulation and identified several miRNA families and RBPs that may act as *cis*-regulators. Our results extend previous observations of the dynamics of APA in primary murine T cells and suggest the presence of additional levels of plasticity in the processes controlling 3′-UTR alterations allowing the return to a steady-state level of global transcript length.

## RESULTS

### Analysis of division-dependent 3′-UTR length changes in murine CD4^+^ T cells

**FIGURE 1.**
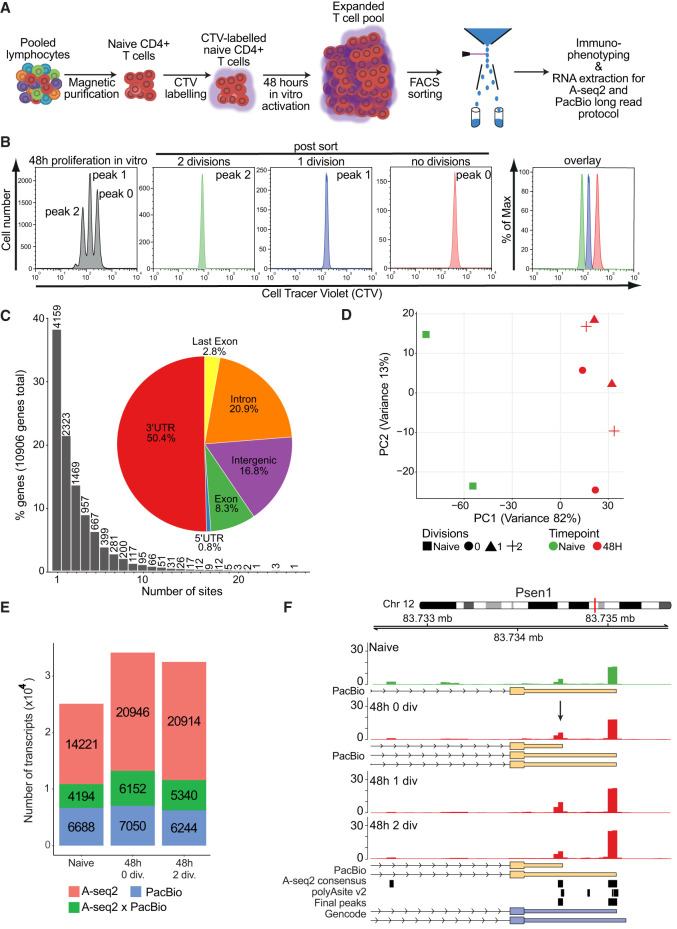
Analysis of division-dependent global 3′-UTR changes in murine CD4**^+^** T cells. (*A*) Schematic workflow of T cell-labeling, activation, and sorting. (*B*) Representative CTV-labeling profile of CD4**^+^** T cells after in vitro activation for 48 h and typical results for sorted peaks of undivided T cells (peak 0), and T cells which are divided once (peak 1) and twice (peak 2). The *right* panel shows an overlay of all populations after FACS sorting. (*C*) Barplot representing the distribution of APA sites per gene. Pie plot shows the genomic localization distribution of APA sites. (*D*) PCA resulting from read coverage over the final peak set. (*E*) Stacked barplot summarizing 3′ UTRs identified by A-seq2 only (red), PacBio only (blue), or both A-seq2 and PacBio (green). (*F*) Example of Psen1 (chr12:83,732,374–83,735,700, “+”) gene 3′-UTR region. Overlap of A-seq2 peaks (consensus) and PolyASite database is labeled as the “Final peaks” track. A-seq2 signal of the different sorted cell populations is represented. Yellow boxes represent isoforms identified using PacBio long-read sequencing (arrows indicate newly identified isoforms), blue boxes represent Gencode (v25) isoforms, and black boxes indicate PAS.

Cancer cell lines and proliferating primary cells have been reported to exhibit APA events with global 3′-UTR shortening (Sandberg et al. 2008; Gruber et al. 2016). To further investigate the relationship between proliferation, differentiation, and APA, we determined the changes of the 3′-UTR landscape during the response of murine primary naive CD4^+^ T cells to stimulation. We purified naive CD4^+^CD62L^−^ T cells and labeled the cells with a fluorescent dye (CellTrace Violet, CTV), which allows the tracking of the number of divisions as the fluorescence intensity becomes twofold smaller with each division. Cells were stimulated through anti-CD3/anti-CD28 antibody cross-linking of the TCR in vitro leading to activation and subsequent proliferation (Fig. 1A). After 48 h, the activated T cells were sorted to high purity (>98%) according to the dye intensity peaks that resulted in three distinct populations: T cells that did not divide yet (peak 0), cells that have divided once (peak 1), and cells that have divided twice (peak 2) (Fig. 1B). All sorted populations were used for RNA extraction (Fig. 1A,B). A fourth population consisted in a CTV-labelled naive CD4^+^ T population (unstimulated) which in parallel was subjected to TRIzol-based RNA extraction at the initiation of the proliferation experiment. We analyzed the expression of the activation markers CD44, CD62L, CD69, and CD25 on all sorted populations of divided T cells. Each division was characterized by a distinct expression of these markers. Cells in peak 0 expressed the lowest levels of CD44 and displayed heterogeneous expression of CD69 and the high-affinity IL-2 receptor CD25. Cells in peak 1 uniformly expressed high levels of the early activation markers CD69 and CD25. Cells in peak 2 showed the highest expression of CD25 and CD44, whereas CD69 expression was lower than in peak 1. Thus, T cells within peaks 1 and 2 underwent full activation, whereas T cells within peak 0 consisted of a more heterogeneous population. Most cells had up-regulated CD69 and CD25, but the activation did not result in cell division (yet), whereas other cells had not induced CD69 expression (Supplemental Fig. S1A,B). To monitor changes in the global 3′-UTR landscape of naive, activated and proliferating T cells with a division-dependent resolution, we prepared 3′ end sequencing libraries from all four populations using the previously established A-seq2 protocol (Gruber et al. 2016; Martin et al. 2017). To identify A-seq2 read enriched regions, or “peaks,” genome-wide, we used a density-estimation approach (Boyle et al. 2008). We retained a peak if it was present in both technical replicates and in at least one biological sample. These peaks were then filtered with poly(A) sites available in the PolyASite database (Herrmann et al. 2019). In total, we retained 43,311 peaks, distributed on 10,906 genes. For 38.1% of the genes (4159), we identified one site (Fig. 1C), thus these genes were not experiencing alternative 3′-UTR polyadenylation in our model. Nearly half of the A-seq2 peaks (50.4%) were located in 3′ UTRs, the remaining being distributed across introns, exons, last exons, 5′ UTR, and noncoding regions (20.9%, 8.3%, 2.8%, 0.8%, and 16.8%, respectively). A principal component analysis (PCA) using the log-transformed RPM (reads per million mapped reads) of reads located on A-seq2 peaks showed that a high proportion of the variance was explained by activation, along the first principal component (PC1) (Fig. 1D). Focusing on activated samples, we observed higher variance between no division (peak 0) T cell samples and one and two divisions samples (peaks 1 and 2, respectively) than between samples from one or two divisions (Supplemental Fig. S1C). In addition, with the goal to generate a qualitative catalog of transcripts effectively present, we used a low coverage long-read PacBio sequencing from naive and 48 h activated T cells (no division and two divisions). In line with what was observed with the A-seq2 protocol, more than half of the covered genes presented one isoform (Supplemental Fig. S1D); 4194, 6152, and 5340 3′ ends (in the naive, zero, and two divisions samples, respectively) colocalized with 3′ ends of transcripts identified by long-read sequencing, whereas 6688, 7050, and 6244 were only identified with long-read sequencing. Conversely, 14,221, 20,246, and 20,914 3′-UTR ends were only identified by A-seq2 (Fig. 1E). Due to limited coverage, while these data showed highly promising value, they did not cover the whole transcriptome and were only used as an additional informative layer, in particular, for visualization (Supplemental Table S1). We then compared our 3′ end sequencing data to a similar study performed by Hsin et al. (2018). They analyzed the effect of miR-155-deficiency in different murine cellular contexts, including CD4^+^ T cells, through differential iCLIP, RNA-seq, and 3′ UTR-usage analysis with poly(A)-seq. The authors compared naive CD4^+^ T cells and in vitro activated CD4^+^ T cells stimulated for 24 h and 48 h. Although the experimental setups were not identical—Hsin et al. (2018) did not sort 48 h activated cells according to cell divisions, and the poly(A)-seq protocol they used differs slightly from the A-seq2 protocol—the covered genes and genomic distribution was comparable (Supplemental Fig. S1E). Similar important activation effects, when comparing naive to 48 h activated cells, have also been observed by analyzing the transcriptome (Dölz et al. 2022). Finally, we observed an equivalent distribution of the variance when reanalyzing the poly(A)-seq data generated by Hsin and colleagues (Supplemental Fig. S1F). We illustrate the data we obtained for a specific gene, Psen1 (Fig. 1F). This example shows how PacBio long-read sequencing can identify novel transcripts, which are not in the reference annotation (Gencode v25), and how well the 3′ end of the long read colocalized with the 3′ end revealed by A-seq2. Using our newly generated 3′ end sequencing data, we observed a clear effect of CD4^+^ T cell activation on 3′ end coverage. We also observed differences between 48 h activated no division cells and 48 h activated cells after one and two divisions. Identification of more than one putative 3′ end in ∼60% of genes with A-seq2 signal showed the diversity of transcript abundances during activation and proliferation phases.

### Identification of alternative poly(A) site usage

**FIGURE 2.**
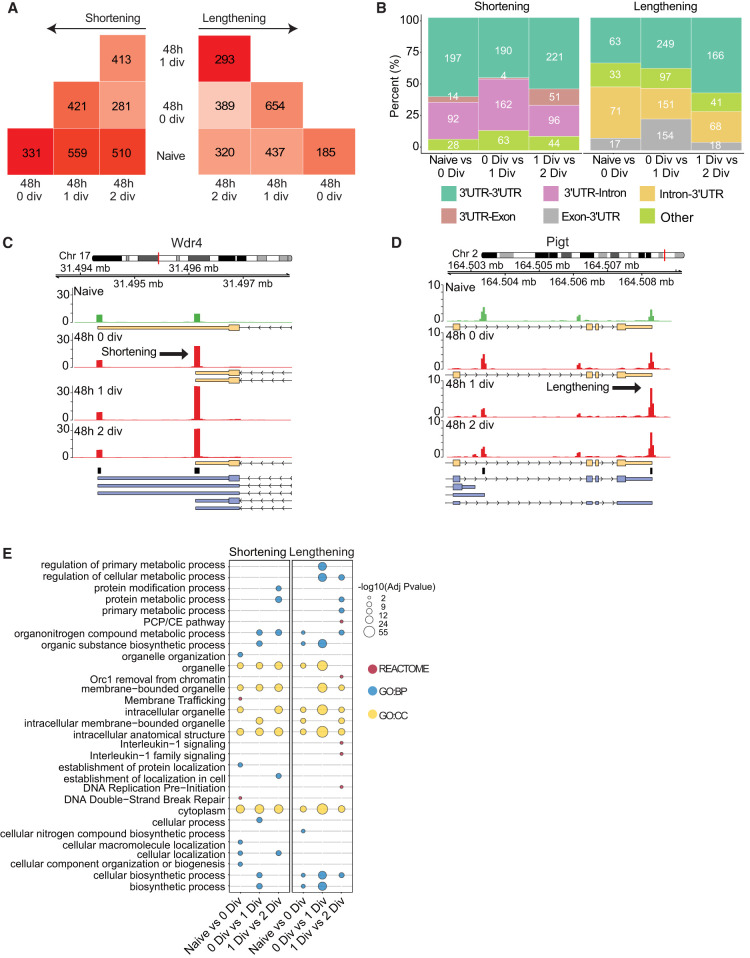
Identification of alternative poly(A) site usage. (*A*) Number of shortening and lengthening events identified in each comparison. Dark color indicates higher numbers, light color indicates lower numbers. (*B*) Classification of APA events per type of transition. (*C*,*D*) Example of Wdr4 gene (chr17:31,493,503–31,497,899, “−”), whose shortened transcripts are more abundant upon activation, and Pigt gene (chr2:164,502,304–164,508,853, “+”), whose longer transcripts are more abundant upon activation. Arrow indicates the event direction. Yellow boxes represent isoforms identified using PacBio long-read sequencing, blue boxes represent Gencode (v25) isoforms, and black boxes indicate PAS. (*E*) Functional annotations of genes associated with an APA event. Dot colors indicate source types. Dot size represents −log_10_(adjusted *P-*values).

Changes in 3′-UTR length are typically inferred from significant differences in 3′ end read coverage between different putative poly(A) sites. We decided to use an approach which, at gene-level, uses vector projection and nonnegative matrix factorization and allows the identification of changes in tandem poly(A) sites, but also when more alternative poly(A) sites are located in between most distal and most proximal sites (Yalamanchili et al. 2020). For this analysis, we did not consider intergenic sites but, in addition to UTR sites, we included intronic and exonic poly(A) sites. We devised a pairwise comparison of naive and all activated samples (Fig. 2A). Event types were stringently defined to fulfill two conditions: the shortening event was defined when sample B proximal projection was greater than sample A proximal projection and if sample B distal projection was smaller than sample A distal projection. The opposite conditions defined a lengthening event. While we observed, on average, a higher number of shortening events, in line with work reported previously about CD4^+^ T cell activation and proliferation (Gruber et al. 2014, 2016), we also observed a substantial number of transcripts using more distal APA sites. In addition, a higher number of events were detected when comparing naive to one and two divisions 48 h activated stages (559 and 510 shortening and 437 and 320 lengthening events) than when naive and activated without division stages were compared (331 and 185 for shortening and lengthening events, respectively). The overlap with shortened and lengthened transcripts identified with Hsin and colleagues’ data set was significant (hypergeometric test: *P*-value = 8.7 × 10^−5^ and 1.1 × 10^−9^, respectively). Most of the observed changes were due to APA at tandem poly(A) sites in the same 3′ UTR, followed by events involving intronic and then exonic sites (Fig. 2B). To note, the association between APA and intronic regions was significant in both type of events (shortened transcripts Fisher *P*-value = 4.9 × 10^−15^ [Cramer's *V* = 0.13 {medium association}] and lengthened transcripts Fisher *P*-value = 4.6 × 10^−11^ [Cramer's *V* = 0.1 {medium association}]). As an example, the short *Wdr4* isoform, whose gene encodes a WD repeat protein required for 7-methylguanosine modification of tRNAs (Alexandrov et al. 2002), became more prominent upon activation (Fig. 2C). One of the most proximal sites in this gene accumulated significantly more A-seq2 reads in samples from activated cells, suggesting a higher abundance of the shorter transcript isoform compared to the longer isoform. In contrast, *Pigt*, coding for a protein involved in glycosylphosphatidylinositol (GPI)-anchor biosynthesis (Ohishi et al. 2001), was expressed as the longer isoform after activation, especially when comparing naive to one division activated samples, through the increased use of a more distal site, even though the shorter isoform remained detectable (Fig. 2D). In that example, the shortest isoforms were not identified with PacBio data. The S100bp gene showed an increased use of its most distal site, the long 3′-UTR isoform remaining dominant (Supplemental Fig. S2). Functional annotation (Fig. 2E; Supplemental Fig. S3A) showed, overall, the enrichment of APA transcripts in the organelle, membrane trafficking pathways, or intracellular transport, suggesting a role for APA in cellular transcript localization through possible regulation of their exposure to cofactors such as RBPs. We further noticed the enrichment of transcripts with lengthened 3′ UTRs in the IL-1 pathway, which was observed only in the transition between one and two divisions. Finally, we also observed the shortening and lengthening of 3′ UTRs of transcripts associated with metabolism only between one and two divisions in activated samples supporting the notion that the metabolism keeps changing upon cell proliferation, but division is needed to initiate some 3′-UTR shortening/lengthening events.

### Dynamic of alternative poly(A) site usage

**FIGURE 3.**
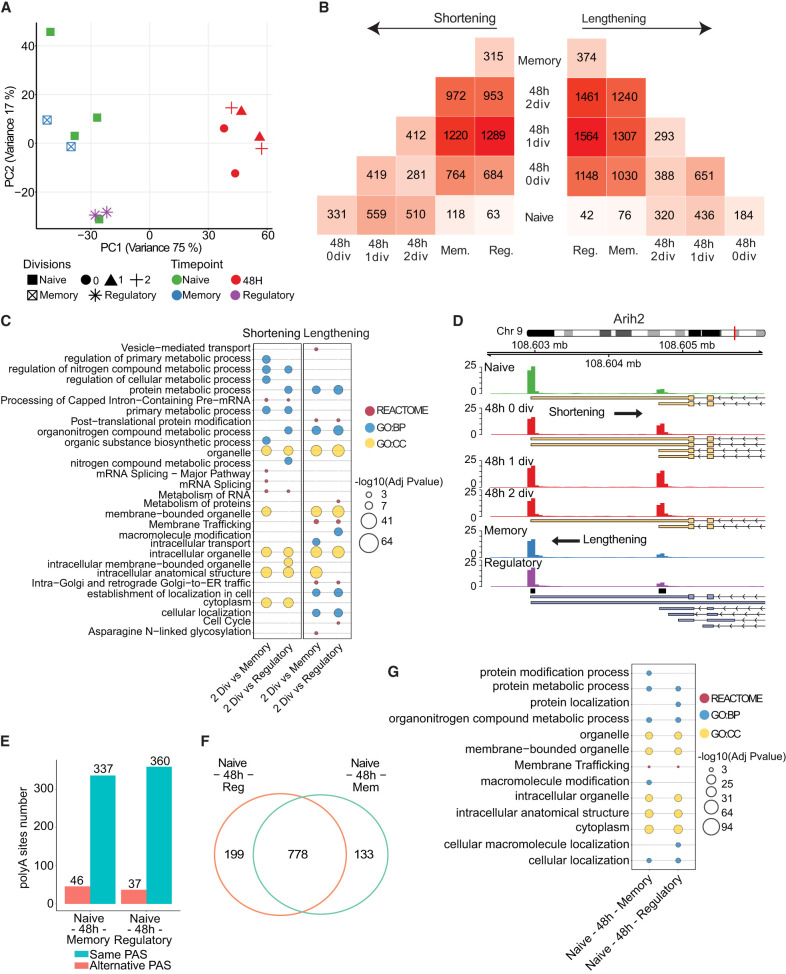
Dynamic of alternative poly(A) site usage. (*A*) PCA resulting from A-seq2 read coverage over the final peak set using naive, all 48 h activated, memory, and regulatory T cells. (*B*) Number of shortening and lengthening events identified in each comparison. Dark color indicates higher numbers, light color indicates lower numbers. (*C*) Functional annotations of genes associated to an APA event between activated two divisions and either memory or regulatory states. (*D*) Example of 3′-UTR Arih2 gene (chr9:108,602,316–108,606,119, “−”) showing reverted length during activation and effector stages. (*E*) Concordance of APA sites’ usage in genes associated with a reverting event. (*F*) Venn diagram showing overlap of genes presenting a reverting pattern. (*G*) Functional annotations of genes associated with a reverted APA event.

While our and previous data sets demonstrate that global changes in 3′-UTR length occur in T cells that are activated and dividing in vitro (Sandberg et al. 2008; Gruber et al. 2016), it is yet unknown in which T cell subpopulations these changes occur. When naive T cells are activated, they enter a phase of massive proliferation (clonal expansion). At the end of the immune response, the clonal population collapses (attrition), leaving a memory population of more quiescent T cells. We thus sought to determine how the polyadenylation landscape is remodeled as cells exit proliferation and become memory T cells. To this end, we sorted three different T cell populations from C57BL/6 mice: naive T cells (CD4^+^ CD25^−^ CD62L^+^ CD44^−^), regulatory T cells (CD4^+^ CD25^+^), and effector memory T cells (CD4^+^ CD25^−^ CD62L^−^ CD44^+^) (Supplemental Fig. S4A). After RNA extraction, we generated a second batch of 3′ ends library by A-seq2 and compared them with the first batch (Fig. 3A). The batch effect was limited, the variance within the four naive samples was low (<8%) and therefore, we did not correct the data for batch effect and considered all naive samples as replicates. PCA using RPM normalized read count over poly(A) sites indicated that CD4^+^ regulatory and memory T cells were more similar to naive CD4^+^ T cells compared to 48 h activated CD4^+^ T cells. The comparison of significant APA event numbers (false discovery rate [FDR] < 0.05) reflected the relationships observed in the PCA (Fig. 3B): Differences in poly(A) site usage were smaller between naive and memory or regulatory T cells than between either of these and activated T cells. This was the case for both, shortening and lengthening, respectively. We annotated genes in which an APA event occurred between the second division following activation and acquisition of a memory and regulatory T cell phenotype (Fig. 3C; Supplemental Fig. S3B).

Organelle-related pathways were enriched in both shortened and lengthened transcripts in these comparisons suggesting intense membrane trafficking changes as observed before (Grumont et al. 2004). Some pathways were specific to shortening events (e.g., Th1 and Th2 cell differentiation, ubiquitin proteolysis, or metabolism of RNA), whereas others were specific to lengthening events (e.g., vesicle-mediated transport, endocytosis, Mapk signaling pathway). Thus, our data suggested that 3′-UTR modulation may differentially affect various T cell subsets, an observation in line with the notion that APA is context-dependent (Mitschka and Mayr 2022).

To illustrate the dynamics of APA on a specific gene, we chose the Ariadne RBR E3 Ubiquitin Protein Ligase 2 (*Arih2*) whose hematopoietic deficiency has been shown to cause lethal activation of the immune system (Lin et al. 2013) because it was associated with a reversible remodeling of 3′ UTRs (Fig. 3D). At the level of the entire transcriptome, in 87% of the cases, the dynamic of 3′ UTR shortening and lengthening involved the same APA sites (Fig. 3E). Transcripts with reversible 3′-UTR usage in either memory or regulatory T cell differentiation (Supplemental Fig. S4B,C) were very similar (778 transcripts of 1110) between these cell populations (Fig. 3F); 199 and 133 transcripts underwent 3′-UTR length changes specifically in one of the two cell populations (regulatory and memory CD4^+^ T cells). We functionally annotated those specific genes (Fig. 3G; Supplemental Fig. S3C). Similarly to memory T cells, regulatory T cells showed enrichment in organelle organization. Finally, we analyzed and interrogated scRNA-seq data about the reversibility of poly(A) site usage. We selected five scRNA-seq data sets from four different studies (Supplemental Fig. S5A,B; Materials and Methods). Cells were annotated using gating models based on transcriptomic expression (Supplemental Fig. S5C). After quality assessment and annotation, we removed one study due to a very limited number of relevant CD4 T cells annotated (either naive, activated, regulatory, or memory). We identified 2530 naive, 2674 activated, 1662 regulatory, and 1451 memory CD4 T cells (Supplemental Fig. S5D). We used SCUREL (Burri and Zavolan 2021) to identify APA at the single-cell level (Supplemental Fig. S5E). One hundred and eighty-eight genes presented a reverted pattern. The overlap with patterned genes identified by using A-seq2 (*n* = 1110) was not significant (Supplemental Fig. S5F), but this analysis showed that reversibility of poly(A) site usage was validated by other data sets where the biological conditions (in particular the CD4 T cell activation conditions) were different. Altogether, these data showed that poly(A) site usage changes transiently during CD4^+^ T cells activation, proliferation, and differentiation, and is associated with specific functions during those phases.

### APA events and transcript abundance regulation

**FIGURE 4.**
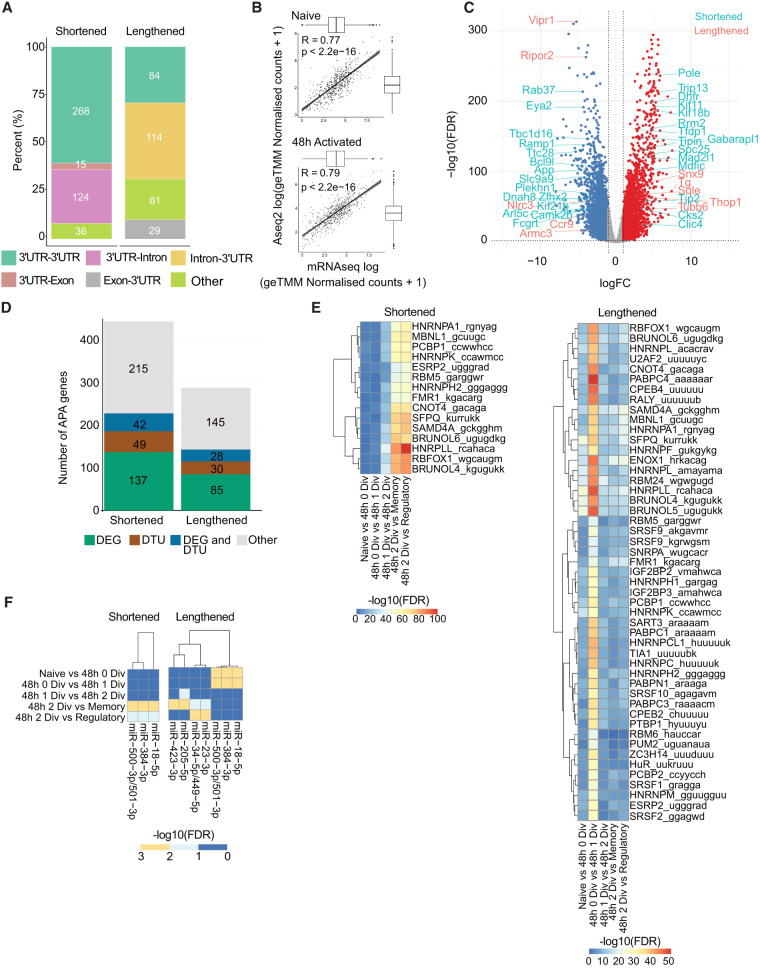
APA events and transcript abundance regulation. (*A*) Classification of APA events per type of transition. (*B*) Pearson correlation of mRNA-seq (Dölz et al. 2022) and A-seq2 gene coverage at naive (*left*) and 48 h after activation (*right*). Reported values are log-transformed normalized counts + 1. Boxplots indicate value distribution and their mean. (*C*) Volcano plot resulting from mRNA differential analysis (Dölz et al. 2022). Blue and red dots indicate genes with FDR < 0.01 and absolute logFC > 1. APA genes that are also DEG are indicated: light blue indicates shortening event and red, lengthening event. (*D*) Barplot reporting number of APA genes is also differentially expressed (DEG), having a DTU of both or none. (*E*,*F*) Heatmaps show enrichment for miRNA (*E*) and RBP (*F*) binding sites within alternative 3′ UTR by type of event, shortening or lengthening. Color legend indicates −log_10_ (FDR).

While many recent studies have focused on the mechanisms regulating the choice of poly(A) sites (Bucheli et al. 2007; Larochelle et al. 2017; Gruber et al. 2018; Grassi et al. 2019), decoupling transcriptional and posttranscriptional effects in the regulation of isoform abundance remains a key challenge. We interrogated our data set by comparing it to a previously generated orthogonal total mRNA sequencing data set generated from naive and 48 h activated CD4^+^ T cells (Dölz et al. 2022). While the activation period of this data set matched with our study, it did not discriminate cells by division. To mimic this, we merged reads of all 48 h activated A-seq2 samples and created four pseudoreplicates, accordingly with naive samples (see Materials and Methods). We first identified genes associated with an event by comparing naive and all 48 h activated samples (Fig. 4A). We identified more shortening than lengthening events: 443 shortening events and 288 lengthening events. Similarly to the first analysis (Fig. 2B), the majority of events involved sites located in 3′ UTR or in intronic regions. Estimated levels of gene expression from A-seq2 and mRNA-seq data were overall well correlated for naive T cells and 48 h after activation (Spearman *r* = 0.77; *P* < 2.2 × 10^−16^ in naive and *r* = 0.79; *P* < 2.2 × 10^−16^ in activated samples) (Fig. 4B). These results were in line with the observation that APA usage estimates from these two approaches correlate enough to be used by bulk RNA-seq data based tools (Ha et al. 2018; Ye et al. 2018; Wang and Tian 2020; Gerber et al. 2021). To annotate transcriptional changes in genes that also exhibited APA, we performed a differential gene expression and a differential transcript usage (DTU) analysis using the mRNA-seq data (Fig. 4C). Forty-eight hours post activation, we observed an important rewiring of the transcriptome: 5659 genes were differentially expressed (DEG) (FDR < 0.01; absolute log fold change [FC] > 1; Supplemental Table S3). Of the 443 genes with shortened 3′ UTRs, 137 were also DEG (31%), 49 were associated with DTU (11%), and 42 (9%) were both DEG and DTU. Of the 288 genes with lengthened 3′ UTRs, 85 were also DEG (30%), 30 were associated with DTU (10%), and 28 were both DEG and DTU (9%) (Fig. 4D).

Functional annotation of APA genes when comparing naive and all combined activated samples (not taking into account the cell division status) (Supplemental Fig. S6) revealed biological pathways related to organelle biology shared both by APA genes being shortened or lengthened. Of note, the ubiquitin–protein transferase activity pathway was enriched only in lengthened transcripts, whereas the ubiquitin–protein ligase activity pathway only in shortened transcripts.

The adequate abundance and localization of transcripts and proteins, crucial for the cell, are regulated at different levels. Longer transcript isoforms can be more exposed to posttranscriptional regulation because they can contain more *cis*-regulatory miRNA (Sandberg et al. 2008) or RBP recognition sites (Jiang and Coller 2012; Gruber and Zavolan 2019). To analyze whether shortening or lengthening events remove or add putative RBP sites, for each comparison we scanned dynamic 3′-UTR regions for RNA motifs known to be recognized by RBPs, all available in the RBPmap database (Paz et al. 2014). Out of the 92 tested motifs, 64 were significantly enriched, in the alternatively included region of either shortened (15) or lengthened 3′ UTRs (49), compared to the 3′-UTR regions of genes that did not exhibit APA in T cells (Fisher's exact test corrected for multitesting; FDR < 0.001) (Fig. 4E). Among the top significant associations, HNRPLL is already known to promote memory T cell RNA rearrangements (Wu et al. 2008), and in the control of alternative splicing in T cells (Gaudreau et al. 2012), ZC3H14 has a known role in controlling the length of poly(A) tails (Kelly et al. 2014). To note, we observed the strongest enrichments in the activated to memory/regulatory comparisons in shortened UTRs, whereas the strongest enrichments were observed after the first division in lengthened UTRs.

We repeated this analysis with a set of well characterized and conserved miRNA binding sites available in the Mouse TargetScan database (McGeary et al. 2019). A total of 3/218 miRNA binding sites were significantly enriched relative to the background (Fisher's exact test; *P*-value < 0.05; the background was genes expressed in CD4**^+^** T cells) in shortened genes, and seven in lengthened genes (Fig. 4F). Among the miRNAs expressed in T cells, we found enrichment for miR-18-5p, in the activated versus memory/T regulatory comparison of shortened genes and in naive to activated samples in lengthened genes, which is part of the miR-17–92 cluster and known to be involved in T cell activation and differentiation (Cho et al. 2016; Pua et al. 2016; Dölz et al. 2022). We also found an enrichment for miR-23-3p which is part of the miR-23–27–24 cluster, known to control effector T cell differentiation and function (Cho et al. 2016). Furthermore, we noted binding sties for several miRNAs which are not yet characterized in T cells (miR-500-3p/501-3p, miR-423-3p). Finally, we investigated DNA-binding transcription factors (TFs) in 3′-UTR regions. Similarly to miRNA and RBPs, we scanned dynamic 3′-UTR regions with TFs binding sites available in the ReMap database (2022) (Hammal et al. 2022). We selected mouse data sets corresponding to CD4-POS biotype (96 data sets, 27 TFs, and 1,179,771 peaks) and found significant enrichment for STAT2 TF (Fisher's exact test corrected for multitesting; FDR < 0.05) both in shortening 3′ UTRs (naive vs. activated no division and activated no division vs. one division) and lengthening 3′ UTRs (activated two divisions vs. memory and regulatory). STAT2 is known to play an important role in the immune response to type 1 interferon signal (Park et al. 2000). While the role of TFs in the choice of alternative poly(A) site has been shown from the enhancer point of view (Kwon et al. 2022), their mode of action at 3′-UTR regions remains elusive. The present analysis suggested a well-balanced system in which APA usage has a limited effect on global gene expression.

## DISCUSSION

**FIGURE 5.**
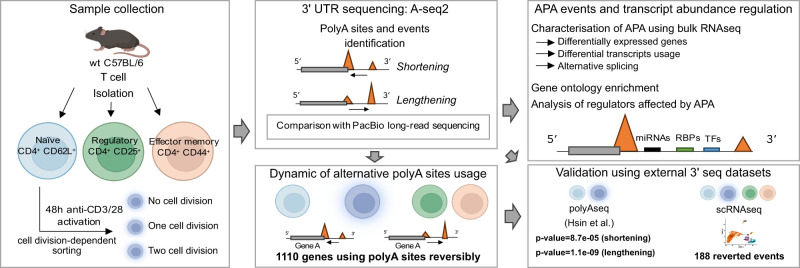
Graphical overview of the analysis.

In this study, we have analyzed the dynamics of 3′-UTR length during T cell activation and differentiation (Fig. 5). While we previously assessed APA in pan-T cells after 72 h of in vitro stimulation (Gruber et al. 2014), here we sought to investigate the 3′-UTR landscape in purified naive CD4^+^ T cells and to compare it to 48 h in vitro activated CD4^+^ T cells as well as ex vivo purified effector memory and Treg cells. In addition, we aimed to resolve the effects on APA induced by T cell activation versus proliferation. To this end, we used a division-dependent resolution and we combined direct detection of 3′ ends using A-seq2 with PacBio long-read sequencing. This allowed us to better define the 3′-UTR dynamics, associated with particular posttranscriptional regulators. We observed that, on average, there were slightly more 3′-UTR shortening events than 3′-UTR lengthening, in line with previous work (Hsin et al. 2018), but different from what was observed upon sustained T cell activation (Sandberg et al. 2008). Our analysis of effector memory T cells and Treg cells suggests that APA is transient since the 3′-UTR site usage of these cells was more similar to naive T cells than activated T cells. While the activation and subsequent proliferation of naive CD4^+^ T cells resulted in global changes in the 3′-UTR landscape, we were interested in the steady-state situation of distinct T cell populations. By looking at the 3′-UTR landscapes of naive and memory T cells, we were comparing a polyclonal pre- to a postexpansion population. One limitation of this study comes from the zero division activated cells which were phenotypically heterogeneous. It might therefore be that the number of APA genes would increase with stronger activation. Thus, our data do not allow a thorough conclusion to differentiate the effect of T cell activation versus cell cycle entry. Nevertheless, by pairwise comparison of cell division stages, we could show an unprecedented APA dynamic usage at the cell division level. Our data also suggest that effector/memory and regulatory CD4**^+^** T cells shared poly(A) sites usage over naive CD4 T cells and point to a yet unrecognized reversibility of global 3′-UTR dynamic. APA therefore exhibits a form of plasticity that is inducible upon T cell activation and might be reversible in vivo after differentiation into memory cells. Furthermore, we also observed this phenomenon using published scRNA-seq data sets. However, the identified reverted APA in scRNA-seq did not overlap significantly with our A-seq2 reverted APA. This may be explained by technical (sparsity) and/or biological (different types of activation in particular) bias and limitations (annotation step) encountered by every scRNA-seq data. In contrast, when comparing APA genes with an external data set using very similar activation conditions (Hsin et al. 2018) but a different method, the overlaps were significant. Comparisons with published data using an mRNA data set showed that APA genes were also differentially expressed between naive and activated samples, and ∼10% presented a DTU, demonstrating a marginal effect or association of APA with global gene expression and transcript usage. Importantly, APA does not equally affect all transcripts (Gruber et al. 2016). It has been shown that miRNAs and RBPs coevolved with APA events (Karginov et al. 2022), resulting in an intricate balance of regulation versus counter-regulation to control cell fate decisions. The availability of 3′-UTR binding sites on specific transcripts and the differential expression of miRNAs and RBPs participate in the competition between different regulators of mRNA abundance supporting a regulatory network that might serve as a posttranscriptional determinant of cellular function. A potential role of DNA-binding TFs localized on 3′-UTR regions is also a promising angle of research, in particular, their role as protein recruiters.

Future work on transcriptional regulation in different T cell types and differentiation stages, but also B cell populations, is needed to reveal the involvement of APA within tightly regulated differentiation steps and receptor activation thresholds controlled in all ranks to ensure a balanced and working immune system. Technology-wise, approaches such as nascent-RNA sequencing coupled with 3′ end sequencing at the single-cell level will help to answer unsolved questions.

## MATERIALS AND METHODS

### T cell isolation, stimulation, and sorting

Spleens and lymph nodes were isolated from male C57BL/6N mice at an age of 6–10 wk. Animal work was done in accordance with the federal and cantonal laws of Switzerland. All organs were collected on ice in complete T cell medium (RPMI 1640 [Sigma], 10% heat-inactivated FCS [Atlanta Biologicals], 2 mM Glutamax [Gibco], 50 μM β-mercaptoethanol [Gibco], 10 mM HEPES [Sigma], and nonessential amino acids [Gibco]), and organs were mashed with a syringe plunger through 45 µm filters in medium under sterile conditions. Cell suspensions were transferred to 15 mL tubes and centrifuged for 5 min at 400*g*, 4°C. Red blood cell lysis was performed with ACK buffer for 5 min at RT and stopped by the addition of a medium. Naive CD4^+^ T cells were isolated with the EasySep Mouse Naive CD4^+^ T Cell Isolation Kit (STEMCELL Technologies) according to the manufacturer's recommendations. For naive T cell activation, 12-well plates (Corning) were coated with 1 mL monoclonal anti-CD3 (2 µg/mL, 2C11, Bio X Cell) in 500 µL PBS for 2 h at 37°C, 5% CO_2_. Purified naive CD4^+^ T cells were labeled with 2 µL CellTrace Violet dye (CTV; in DMSO) in 2 mL PBS for 20 min at 37°C, and the labeling reaction was stopped by the addition of 10 mL complete medium. Cells were plated in a density of 4 × 10^6^ CTV-labeled naive T cells per coated 12-well or six-well plates in 2 mL or 4 mL, respectively, plus anti-CD28 antibodies (1 µg/mL, PV-1, Bio X Cell) and incubated at 37°C with 5% CO_2_ for 48 h. A total of 4–6 × 10^6^ purified naive CD4^+^ T cells were resuspended in TRIzol on the day of T cell purification (day 0). After 48 h of cell culture, cells were harvested and stained with 1 µg/mL propidium iodine briefly before sorting. Cells were sorted by their distinct proliferation peaks (peak 0, peak 1, and peak 2) on FACS-Aria and Influx Cell Sorters (BD Biosciences). Cells were kept on ice and after washing with cold PBS were immediately resuspended in TRIzol for RNA extraction. One portion of the cells was used for surface phenotype staining with the fluorochrome-conjugated mAbs: anti-CD4 (clone RM4-5), anti-CD25 (PC61), CD62L (MEL-14), CD44 (IM7), CD69 (H1.2F3), CD16/32 (93, all BioLegend). Cells were measured on an LSR-Fortessa (BD Biosciences), and data were analyzed using FlowJo (TreeStar). For isolation of steady-state populations of CD4^+^ naive, memory and regulatory T cells, cells were pre-isolated with the EasySep Mouse CD4^+^ T Cell Isolation Kit (STEMCELL Technologies) and stained with the fluorochrome-conjugated mAbs, targeting CD4, CD44, CD62L, and CD25 before being sorted on FACS-Aria and Influx Cell Sorters (BD Biosciences). Purified populations were washed with cold PBS and immediately resuspended in TRIzol for subsequent RNA extraction.

### RNA extraction

Isolated cells were quickly processed and washed with cold PBS before resuspension in 1 mL TRIzol. Samples were left at room temperature for 5 min and frozen at −20°C. For RNA extraction, TRIzol samples were thawed on ice, and 100 µL 1-Bromo-3-Chloropropane was added before mixing and incubation for 15 min at RT. Samples were centrifuged for 15 min at 12,000*g*, 4°C, and the aqueous phase was transferred to a fresh tube before mixing with 1 volume of isopropanol and a subsequent incubation of 10 min at RT. Samples were then centrifuged for 15 min at 12,000*g*, 4°C, and the RNA pellet was washed with 1 mL ethanol followed by a centrifugation for 30 min at 12,000*g*, 4°C. The supernatant was discarded and the pellets were briefly dried before the RNA was dissolved in dH_2_O and stored at −80°C.

### A-seq2 protocol

Briefly, poly(A)+ RNAs were isolated from RNA samples and fragmented by alkaline hydrolysis. Next, adaptors were ligated to the 5′ end of enriched poly(A)+ RNAs, and reverse transcription was carried out with a bio-dU-dT(25) RT-primer. After USER-enzyme digestion and purification, a second adaptor was ligated to the 5′ ends of the resulting cDNA. Both adaptors were finally used to PCR-amplify libraries with barcoded primers and used for RNA sequencing (Gruber et al. 2016; Martin et al. 2017).

### Preprocessing of A-seq2 libraries

We first performed quality analysis and read trimming of A-seq2 read libraries with fastp (v.0.19.7) and the following parameters: minimum length required (-l) = 15, UMIs were integrated by enabling -U, placed in the read 1 (‐‐umi_loc) = read1 and formed of first seven bases of each read (‐‐umi_len = 7), and finally max *N* number limit was set to 2 (‐‐n_base_limit). Mapping of the trimmed reads on the mm10 genome was achieved with STAR (Dobin et al. 2013) (v.2.7.3) by asking end-to-end alignment (‐‐alignEndsType EndToEnd) and no multimapped reads (‐‐outFilterMultimapNmax 1). Finally, we used UMIs to remove duplicated reads with umi_tools (v.1.0.0) (Smith et al. 2017) *dedup* (‐‐umi-separator=“:” ‐‐extract-umi-method=“read_id” ‐‐method=“directional”).

### Poly(A) site quantification and events identification and functional annotations

#### Poly(A) site quantification and genomic annotation

Read enriched regions (peaks) were called using Fseq (v.1.84) (Boyle et al. 2008) with fragment size (-f) of 50, feature length (-l) of 150, and threshold (-t) of 6. Bins around peaks with <10 reads were removed to refine peaks. Within each condition, peaks from each replicate were intersected using BEDTools (v.2.27.1) (Quinlan and Hall 2010), and the resulting intersected peak sets were combined into a master set of peaks using DiffBind R package (v.2.16.2) (Ross-Innes et al. 2012) and a minimum overlap of 1 (peaks need to be present in at least one intersected peak set). The master set of peaks was then sloped by 200 bp on both sides and overlapped with poly(A) sites from the PolyASite resource for mouse version r2.0 (https://www.polyasite.unibas.ch/atlas) (Herrmann et al. 2019). We filtered out peaks with a size >2000 bp and <10 bp. A-seq2 reads were quantified using the featureCounts function from the Rsubread package (v.2.4.3) (Liao et al. 2019). Peaks were categorized based on their genomic locations (5′ UTR, 3′ UTR, Exon, Intron, Intergenic) using Gencode VM25 annotations (https://www.gencodegenes.org/mouse/release_M25.html).

### Event identification and filtering

To identify events (shortening or lengthening), we used PolyA-miner (https://github.com/LiuzLab/PolyA-miner) (Yalamanchili et al. 2020). Parameters were set as follows: poly(A) annotations file (-pa) contained the sites identified above, reference fasta sequence (-fasta) was downloaded from Ensembl (http://ftp.ensembl.org/pub/release-94/fasta/mus_musculus/dna/Mus_musculus.GRCm38.dna.toplevel.fa.gz), and reference genes bed file (-bed) was created from Ensembl gff3 file (v94). The types of events were stringently defined to fulfill two conditions: transcript is shortened if sample B proximal projection was greater than sample A proximal projection and if sample B distal projection was smaller than sample A distal projection. The opposite conditions identified as a lengthened gene. Events were then filtered on FDR (5%). For each pairwise comparison, the MaxAPASwitch position from PolyA-miner output was retained as the most used position on condition B, whereas the most covered position was considered as the most used APA site in condition A. This was done according to type of event and strand. The DNA sequence between sites from conditions A and B was further used for miRNA and RBP binding/motif analysis.

### Functional annotation of APA genes

Functional annotation was carried out using the gprofiler2 R package (v.0.2.0) (Raudvere et al. 2019), the model organism was set to “mmusculus,” “fdr” correction method, and the following sources were interrogated: GO:BP, GO:MF, GO:CC, KEGG, REAC, MIRNA, CORUM, and WP. A 5% FDR threshold was applied and the top five annotations within each of the sources were plotted.

### mRNA-seq processing and analysis

mRNA-seq data count tables were downloaded from GSE140568 and processed similarly to Dölz et al. (2022). Briefly, here are the main steps. The GTF file used along the processing steps was extracted from Gencode VM25.

#### mRNA acquisition and sequencing

A total of 2.5 × 10^5^ cells were washed with PBS, resuspended in 200 μL TRI Reagent, and RNA was extracted from TRIzol samples with a Zymo Direct-zol kit which includes DNase treatment. RNA quality was assessed with a Fragment Analyzer (Advanced Analytical), and RNA-seq library preparation was performed using the Illumina TruSeq Stranded kit. Sequencing was performed on an Illumina NextSeq 500 machine to produce single-end 76-mers reads. All steps were performed at the Genomics Facility Basel (ETH Zurich).

#### Gene-level quantification

Read quality was assessed with the FastQC tool (v.0.11.5) (http://www.bioinformatics.babraham.ac.uk/projects/fastqc). Reads were mapped to the mouse genome (UCSC version mm10) with STAR (v.2.5.2a) (Dobin et al. 2013) with default parameters, except filtering out reads mapping to more than 10 genomic locations (outFilterMultimapNmax = 10), reporting only one hit in the final alignment for multimappers (outSAMmultNmax = 1), and filtering reads without evidence in the spliced junction table (outFilterType-“BySJout”).

#### Differential analysis

Read alignment quality was evaluated using the qQCReport function of the R Bioconductor package QuasR (v.1.18) (Gaidatzis et al. 2015). Gene expression was quantified using the qCount function of QuasR as the number of reads (5′ ends) overlapping with the exons of each gene assuming an exon union model (using the UCSC knownGenes annotation, downloaded on December 18, 2015). The R Bioconductor Package edgeR (v.3.28) (Robinson et al. 2010) was used for differential gene expression analysis. Between-sample normalization was done using the TMM method. Only genes with CPM (counts per million mapped reads) values more than one in at least four samples (the number of biological replicates) were retained. A generalized linear model including a genotype effect, an activation effect, and a replicate effect (nested within genotype) was fitted to the raw counts (function glmFit), and differential expression was tested using likelihood ratio tests (function glmLRT). *P*-values were adjusted by controlling the FDR (Benjamini–Hochberg method), and genes with an FDR lower than 1% were considered differentially expressed.

#### Differential transcript usage

We used the DRIMSeq R Bioconductor package (v.1.20.0) (Nowicka and Robinson 2016) to perform DTU on mRNA-seq data. First, for each four replicates, in naive and 48 h activated samples, salmon (v.1.5.0) quantification was performed at the transcript level using *quant* function and the following parameters: -l A ‐‐validateMappings ‐‐seqBias ‐‐gcBias ‐‐posBias ‐‐softclip ‐‐numBootstraps 100. A full model comparing naive and activated samples was applied, and post hoc filtering was executed (it improves the FDR and overall FDR control by setting the *P*-values and adjusted *P*-values for transcripts with small per-sample proportion SD to 1). Finally, a stage-wise procedure (stageR R bioconductor package v.1.14.0) was used to adjust *P*-values.

#### miRNA quantification

miRNA-seq data were mapped to the mm10 genome using Bowtie2 and ‐‐very-sensitive-local parameter. UMIs were counted using a custom python script.

### miRNAs and RBPs binding sites analysis

Background regions were 3′-UTR regions of genes considered as expressed in T cells (log_2_[CPM + 1] > 0 in at least naive or 48 h activated).

#### miRNAs binding site enrichment analysis

Genome coordinates of predicted conserved targets were retrieved from the TargetScan Mouse database (release 7.2) (Agarwal et al. 2015). This BED file contains genome (mm10) locations of mouse-predicted (conserved) targets of conserved miRNA families and associated score (context++ score percentile). Enrichment for each individual miRNA family against shortening and lengthening regions from each comparison was computed, and the Fisher exact test *P*-value was reported.

#### RBPs motif enrichment analysis

DNA sequences of dynamic 3′-UTR regions were extracted and converted into RNA sequences. They were scanned with RBPmap (v.1.2) (Paz et al. 2014) RBP motifs, and converted into TRANSFAC format. Motif enrichment analysis was performed with Analysis of Motif Enrichment (AME) from MEME suite (v.5.3.3) (McLeay and Bailey 2010). Parameters were as follows: ‐‐scoring avg ‐‐method fisher ‐‐hit-lo-fraction 0.25 ‐‐evalue-report-threshold 10.0 ‐‐control ‐‐shuffle‐‐ ‐‐kmer 2. An FDR threshold of 0.1% was applied.

#### TFs binding site enrichment analysis

Genome coordinates of TFS binding sites (peaks) were retrieved from the mouse ReMap database (2022) (Hammal et al. 2022). We filtered biotypes by selecting CD4-POS to fit our biological model. Enrichment for each individual TF against shortening and lengthening regions from each comparison was computed, and the Fisher exact test *P*-value was reported.

### PacBio long-read sequencing

Three SMRTbell libraries (naive, 48 h activated zero division, and two divisions) from TRIzol isolated high-quality RNA samples (>500 ng total RNA) were sequenced on a Sequel SMRT Cells 1M for a general survey of full-length isoforms in a transcriptome with moderate to high expression levels. SMRTbell libraries and sequencing were achieved by the Department of Biosystems Science and Engineering—ETH (D-BSSE). Tools from PacBio and Bioconda were used to process these data (https://github.com/PacificBiosciences/pbbioconda). Consensus sequences (.ccs) were generated from our raw subread data by using ccs (‐‐min-rq 0.9). Full-length reads were generated after primer removal and demultiplexing using lima ‐‐ccs. Then we removed poly(A) tails and artificial concatemers with isoseq3 refine (‐‐require-polya ‐‐min-polya-length 20) and clustered consensus sequences to generate transcriptome fasta with isoseq3 cluster (‐‐use-qvs). High-quality full-length polished isoforms were mapped to the genome and collapsed into transcripts based on genomic mapping using pbmm2 align (‐‐preset ISOSEQ). We then used isoseq3 collapse to generate fastq and gff files. Transcripts were annotated using SQANTI3 (v.0.1) (Tardaguila et al. 2018) (‐‐aligner_choice minimap2) and provided a list of mouse poly(A) motifs retrieved from the PolyASite database (https://polyasite.unibas.ch/atlas), transcript abundance from isoseq3 output and CAGE peaks from Fantom5 (https://fantom.gsc.riken.jp/5/datafiles/reprocessed/mm10_latest/extra/CAGE_peaks/).

### Single-cell RNA sequencing

#### Data collection and processing

We collected raw FASTQ files from the following repositories: from ArrayExpress E-MTAB-7311 (3324STDY7421494 and 3324STDY7421495 samples), from SRA PRJNA497439 (SRR8075748 and SRR8075750 samples), PRJNA493233 (SRR7904988 and SRR7904987 samples), and PRJNA529806 (SRR8810689 sample). All samples were processed individually with CellRanger (v.7.1.0; Zheng et al. 2017) with mm10 reference 3.0.0 (Ensembl 93; November 19, 2018). Seurat R package (v.4.3.0.1; Hao et al. 2021) was used to import CellRanger outputs in R and create a Seurat object. We applied the following filters: 1000 < Read number < 30,000; 400 < Feature number < 6000; max mitochondrial read percentage = 8, max ribosomal read percentage = 5; features without variation were also removed. After normalization and feature selection, we evaluated the cell cycle effect by computing S and G2M scores using the CellCycleScoring function. Normalized count matrices were scaled and dimension reduced by PCA. Doublets were identified with doubletFinder_v3 (DoubletFinder R package v.2.0.3; McGinnis et al. 2019) and removed. For more details, the entire code is available at https://gitlab.com/dseyres/dynamic_apa.

#### Gating on the expression profile

We used the scGate R package (v.1.4.1; Andreatta et al. 2022) to annotate cells. We used either predefined models retrieved from scGate_models github repository (https://github.com/carmonalab/scGate_models/tree/dev1/mouse/generic) or defined for this study. Cells annotated by different models were annotated as “Multi” and not used for downstream analysis.

#### APA events identification at the single-cell level

We applied the SCUREL method (Burri and Zavolan 2021) to identify shortened and lengthened transcripts among the different comparisons. We did not retain the PRJNA497439 samples because too few cells of interest were annotated. We applied default parameters; except for AUC analysis we set the two-sided probability tail for the significance (α) threshold to 5%.

## DATA DEPOSITION

Data sets have been submitted to GEO under GSE209604 SuperSeries. The A-seq2 data sets generated in this study are available under accession ID GSE183424. PacBio long-read sequencing data are accessible under accession ID GSE209603. The mRNA-seq data set (Dölz et al. 2022) is available under accession ID GSE140568. Code and all necessary material to reproduce this analysis are available on GitLab https://gitlab.com/dseyres/dynamic_apa and on Zenodo (https://doi.org/10.5281/zenodo.10137241). More additional tables and R objects are also provided in this repository, such as DU transcripts, posttranscriptionally and transcriptionally regulated genes, MiRs and RBP occurrences or PolyA-miner raw outputs.

## SUPPLEMENTAL MATERIAL

Supplemental material is available for this article.

## COMPETING INTEREST STATEMENT

L.T.J. is a cofounder and board member of and holds equity in Cimeio Therapeutics AG, a biotech company developing engineered cellular therapies. L.T.J.’s activities related to Cimeio are unrelated to this study. All other authors have no competing interests to declare.

## Figures and Tables

**Denis Seyres:**
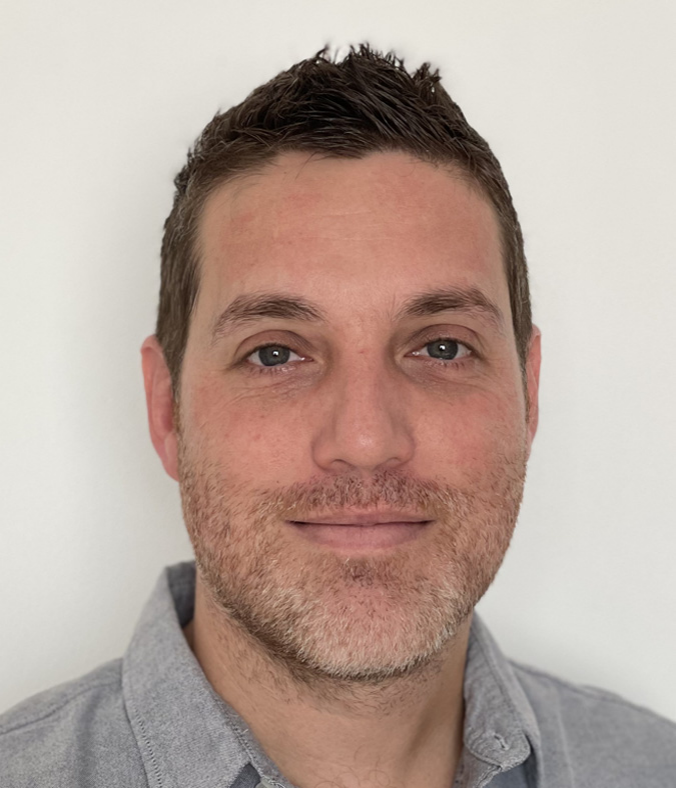


**Oliver Gorka:**
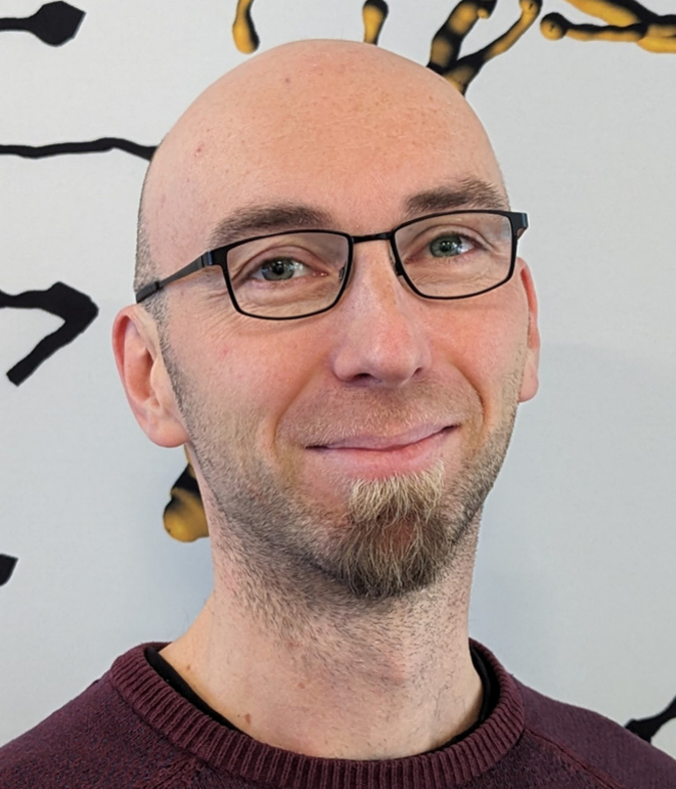


## MEET THE FIRST AUTHORS

***Meet the First Author(s)* is an editorial feature within *RNA*, in which the first author(s) of research-based papers in each issue have the opportunity to introduce themselves and their work to readers of *RNA* and the RNA research community. Denis Seyres and Oliver Gorka are the first authors of this paper, “T helper cells exhibit a dynamic and reversible 3′-UTR landscape.” Denis is a computational biologist in Professor Lukas Jeker's lab at the University of Basel. His favorite research field is gene regulation using all possible omics data. Oliver Gorka was a postdoc with Professor Jeker at the Department of Biomedicine Basel, Switzerland at the time of this study. Oliver is currently working as a staff scientist/laboratory manager at the Institute of Neuropathology of the University Medical Center Freiburg, Germany, focusing on inflammasome biology, immune cell signaling, and metabolism.**

**What are the major results described in your paper and how do they impact this branch of the field?**

In our paper we show that poly(A) site usage in T cells is a dynamic, transient, and reversible phenomenon. The A-seq2 results we obtained from our different T cell subsets and conditions were exciting, as the RNA molecules were sequenced through the 3′ end, giving sufficient coverage to confidently call APA sites. For analysis, we applied a method using vector projections and nonnegative matrix factorization (PolyA-miner), which takes into consideration all the possible poly(A) sites, rather than just comparing tandem sites. Interpretation of the results was not trivial, but it made a lot of sense. Another important point was the minor contribution of APA to global gene expression, which suggests a complex transcriptional/posttranscriptional regulation of the different isoforms to maintain the same gene expression level (as seen with RNA sequencing). Looking at the biology of T cells, the APA dynamic seems to follow the dynamic life of a T cell clone: A naive T cell shows alternative poly(A) site usage in comparison to its quiescent steady state once it gets activated and proliferates. After an expansion phase, the pool of T cells collapses, and a cellular memory T response is established. Until a re-encounter of the cognate antigen, this population remains again quiescent along with a reverted poly(A) site usage.

**What led you to study RNA or this aspect of RNA science?**

**D.S.:** As part of my global interest, which is understanding gene regulation, I wanted to learn more about transcriptional and posttranscriptional regulation layers, in particular through the action of miRNAs and RNA-binding proteins. These A-seq2 plus long-read sequencing data were a great opportunity for me to jump in. In the laboratory, we also published matching bulk RNA-seq data, so it was a good occasion to pile up this information.

**O.G.:** During my PhD studies at the Technical University Munich, I studied molecular mechanisms of T cell activation and differentiation in the laboratory of Professor Ruland. One of my projects dealt with Malt1 paracaspase, a critical signaling protein during immune cell activation that in addition cleaves RNA-binding proteins Regnase-1 and Roquin for posttranscriptional control of distinct mRNA species. Molecular regulation mechanisms were always exciting to me, but here I became aware of a new and exciting layer of regulation. To dive deeper into molecular immune regulation on the RNA level, I joined the laboratory of Professor Jeker to work on 3′-UTR dynamics in T cells.

**During the course of these experiments, were there any surprising results or particular difficulties that altered your thinking and subsequent focus?**

**D.S.:** One particular aspect was how to interpret events. For example, a shortening event does not always mean that the shortest isoform is the most abundant. It means that there is a trend to use the shortest isoforms in condition B compared to condition A. It can be counterintuitive, but I think it reflects well the complexity of gene regulation.

**O.G.:** When generating the first A-Seq2 libraries, we were not entirely sure if the input RNA would be sufficient, as we were working in part with limited cell numbers after cell sorting. However, it soon turned out that the established protocol robustly works also for somewhat smaller input. Once this was settled, I was convinced that the experiments would result in interesting data sets worth following up on.

**What are some of the landmark moments that provoked your interest in science or your development as a scientist?**

**D.S.:** Personally, when in the 2000's the first human genome was sequenced, deciphering the genome and understanding the gene regulatory code were the biggest promises. I found that super-motivating and it is leading my entire research career! After two decades, even if we are getting closer and closer, the conclusion is still the same: It is super complex! But I am convinced that deciphering this code is still key to understanding most (if not all) diseases.

**O.G.:** When first working on lymphocytes as a young student, I was truly amazed by the cellular reactions that could be observed through a simple light microscope once you stimulate them in vitro. These perfectly roundish little cells grow in size, form beautiful clumps, and proliferate at an amazing speed. Looking at them from the outside really made me wonder what intricate molecular processes might be going on in there, and how all this is so tightly regulated to keep our bodies healthy. The complexity of the immune system and its importance for every organism made me choose molecular and cellular immunology as my research discipline.

**If you were able to give one piece of advice to your younger self, what would that be?**

**D.S.:** My advice to my younger self is to embrace your ideas and not let fear or self-doubt hold you back. In my earlier days, I generated insightful concepts but hesitated to express them, or didn't know how to exploit/explore these ideas. I realize the importance of taking my ideas seriously, even if it means stepping out of my comfort zone. Trust in your abilities, face challenges head-on, and don't be afraid of rejection.

**O.G.:** I can only back up Denis’ advice. I would also tell my younger self that science can be tough at times and a healthy degree of confidence is essential to push your ideas forward. Another piece of advice would be to get involved with bioinformatics/data science early on in your career, independent of what life science discipline you are working in.

**What were the strongest aspects of your collaboration as co-first authors?**

**D.S.:** To me, as a computational biologist, collaborating with the person performing the experiment is super important and efficient. Oliver has an incredible biological background about this system, and it was super useful to trigger the analysis.

**O.G.:** This collaborative effort would not have been possible without Denis and Ralf from the Zavolan lab. They were truly essential for the thorough analysis of the generated data sets. I enjoyed the interdisciplinary exchange and working with them very much.
